# Acknowledgment to the Reviewers of *Biomimetics* in 2022

**DOI:** 10.3390/biomimetics8010038

**Published:** 2023-01-16

**Authors:** Biomimetics Editorial Office

**Affiliations:** MDPI AG, St. Alban-Anlage 66, 4052 Basel, Switzerland

High-quality academic publishing is built on rigorous peer review. *Biomimetics* was able to uphold its high standards for published papers due to the outstanding efforts of our reviewers. Thanks to the efforts of our reviewers in 2022, the median time to first decision was 16 days and the median time to publication was 37 days. Regardless of whether the articles they examined were ultimately published, the editors would like to express their appreciation and thank the following reviewers for the time and dedication that they have shown *Biomimetics*:
Aboulnaga, MohsenMa, ZhuoranAdamski, MiroslawMahmood, Muhammad ArifAfonso, FredericoMangano, FrancescoAgarwal, GunjanMannu, AlbertoAgrawal, SumitMaranzana, NicolasAhmed, Montaser S.Marques, CarlosAlexeev, AlexanderMartín-del-Campo, MarcelaAlfano, MarcoMarto, Carlos MiguelAlsaad, Mohamad AliMarvasti-Zadeh, Seyed MojtabaAnesi, AlexandreMatsuda, YukikoArrúa, Eva CarolinaMcCardle, JohnAsahara, HaruyasuMcHenry, MatthewAsghar, ZeeshanMeli, VijaykumarAshkenasy, NuritMencattelli, LorenzoAziz, TariqMernik, MarjanBadraoui, RiadhMeyer, FlorentBaldassari, SaraMichno, AnnaBałdowska-Witos, PatrycjaMilazzo, MarioBarasinski, AnaïsMochi, FedericoBarbaro, KatiaMohamed Yusoff, Mohd ZuhriBastida, AgathaMohamed, Ali WagdyBeismann, HeikeMoiseenko, Konstantin V.Bekiari, VlasoulaMolina, Gustavo FabiánBella, FedericoMonat, JamieBenos, LefterisMonkman, GarethBergamini, AndreaMontoya, CarolinaBi, ShichaoMordak, ArkadiuszBlanquer, Sébastien B. G.Morotomi, TakahikoBociong, KingaMouchet, SébastienBonomo, MatteoMourouzis, PetrosBorcia, RodicaMoussa, TalaBormashenko, EdwardMu, ZhengzhiBoroda, Andrey V.Murtra, Andreu CorominasBostenaru Dan, MariaNair, Sithara S.Botez, RuxandraNasaruddin, Ricca RahmanBougdah, HocineNaudi, KurtBowen, JamesNavab, NassirBroeckhoven, ChrisNgen, Ethel J.Brynjólfsson, SigurðurNiewiarowski, PeterBüscher, Thies HenningNikolova, MariaCalixto, SergioNoh, HeesoCalvo, Adriana M.Nourmohammadi, AmirCao, ChongjingNowak, Jan, KrzysztofCao, Chris YingNucci, LudovicaCarvajal Monroy, Paola L.Oancea, FlorinCassidy, John F.Ogay, VyacheslavChambers, LilyOliver, Jeremie D.Chang, Hsin-I.Ongpeng, JasonChatterjee, AtrouliOtsu, HirotakaChatterjee, KaushikOunjai, PueyChellapurath, MrudulPanariello, LucaChen, Ching-YunPanas, AndrzejChen, ChunjiePandey, Lalit MohanChen, GuangrongPang, MuyeChen, MichaelPantuso, ElviraChen, NairongPapagiannis, GeorgiosChen, RuiPatel, Kapil D.Chen, ShaojuanPatrizia, NittiChen, Wen-ChengPerš, JanezChen, Wen-FanPeters, TerriChen, XiaoyuPetros, MourouzisChen, ZhuofanPetutschnigg, AlexanderChernonosova, VeraPiskur, PawelChetoni, PatriziaPlessis, Anton DuChiang, YingchihPollett, AaronChrcanovic, BrunoPolovic, NatalijaChu, JinkuiPortan, DianaCiapetti, GabrielaPrévôt, MarianneCinquemani, SimonePuppin-Rontani, Regina MariaConcilio, AntonioQaderi, SaeedehConserva, EnricoQin, YuyueCordes, FlorianRączka, WaldemarCorreia, IlidioRainey, JanCorsalini, MassimoRamli, Mohd SyakirinDang, ThoRasoga, OanaDatta, Lakshmi PriyaRatautaite, VilmaDe Oliveira Júnior, Antonio CarlosRejeesh, C RDiaz, EsperanzaReque, Priscilla MagroDíez Díaz, VerónicaResende, RodrigoDinis Gaspar, PedroRivas-Tabares, DavidDong, ZhaomingRivero-Buceta, VirginiaDua, RupakRodríguez López, María IsabelDubashynskaya, NatalliaRodríguez-Archilla, AlbertoDucker, WilliamRodríguez-Lorenzo, LuisDuisit, JérômeRomán-Doval, RamónEgedy, AttilaRomano, DonatoElistratkin, Mikhail Y.Rose, Christopher StewartEl-Wassefy, Noha Abdel MawlaRoth-Nebelsick, AnitaErshad-Langroudi, AmirRoveri, NorbertoEstevadeordal, JordiRubio Alonso, FaustoFan, XudongRuksiriwanich, WarintornFelgueiras, HelenaRusso, ValentinaFernandes, JulianaSaeidi, NazaninFerreira, RodrigoSaeloh, DennapaFischer, NicholasSaleemi, SidraFlammang, PatrickSalehabadi, AliFlapper, Walter J.Samarakoon, BhagyaFoglia, Mario MassimoSancini, GiulioFormoso, PatriziaScapin, GiorgiaFrasco, Manuela F.Schaber, Clemens F.Fudickar, SebastianSchaubroeck, DavidFunari, Marco FrancescoSchulze-Tanzil, Gundula GesineFuschi, PaoloSefat, FarshidGalli, CarloSekiguchi, YuGamompilas, ChaiwutSerres, AlexandreGelli, RitaSharifi, SiminGeng, BiaoShin, Soo YoungGeorge, DanielShoute, Lian C. T.Ghazvinian, AliSikavitsas, VassiliosGizzi, AlessioSingh, NarinderGodeau, GuilhemSingh, Surya PrakashGracia Lostao, Ana IsabelSinibaldi, EdoardoGrazioli, GuillermoSkoulas, EvangelosGristina, RobertoSkwarek, EwaGritli, HassèneSmyk, EmilGuan, XiaoyangSobotka, PiotrHafez, IslamSong, BifengHaugen, Håvard JSong, QiHe, GuangpingSousa, FilipaHe, QiguangSpeck, OlgaHerault, JohannStano, GianniHerr, Christiane M.Stavric, MilenaHirtz, MichaelŠtěpanovská, JanaHobzová, RadkaSubrin, KévinHoffmann, BirgitteSun, JingHolešová, SylvaSutherland, TaraHollenbach, MarcusSuzuki, MotoyaHong, Wei-ChiangSydor, MaciejHornfeck, RüdigerTaboryski, Rafael J.Hsu, Ching-ChiTakahashi, YusukeHu, ChunheTauböck, TobiasHu, FuwenTay, Daniel YiweiHu, JianguoTaylor Buck, NickHumar, MihaTenorio-Alfonso, AdriánInokuchi, TsutomuTeo, Mei YingIslam, Md SaifulThamboo, Julian AjithIzydorczyk, JacekTian, HongmiaoJacobs, ShoshanahŢierean, Mircea HoriaJakšić, ZoranTomašegović, TamaraJanos, RudolfTorre, CarloJantschke, AnneTorres-Lagares, DanielJayaraman, JayakumarTrautz, MartinJelińska, AnnaTribst, João Paulo MandesJesionowski, TeofilTsai, Shiao-WenJiang, HongzhouTsepelev, VladimirJiang, JingTurner, JacobJoão, SilvaUdayanga, DhanushkaJodin, GurvanUllah, MuhammadJung, HeechulVasiliu, Ana-LaviniaKallitsis, JoannisVázquez-Victorio, GenaroKambic, RobertVelasco Ortega, EugenioKang, Young-HoonVishwakarma, VinitaKant, KrishnaWan Mohtar, Wan Abd Al-Qadar ImadKapliński, OlegWang, DehuiKaraoglan, Aslan DenizWang, JianKarayannis, Chris G.Wang, JianxinKaur, GurpreetWang, JikaiKennedy, BrookWang, JinyanKepinska, MartaWang, XiaolongKeshavarz, MeysamWang, ZeKhalid, Mohd Nor AkmalWang, ZuankaiKim, Jwa-YoungWanieck, KristinaKim, Mi-YoungWęglarski, MariuszKim, Yong-IlWilliams, John A.Kłysz, SylwesterWitte, Hartmut F.Kohri, MichinariWolanśki, WojciechKorobiichuk, IgorWongchoosuk, ChatchawalKörstgens, VolkerWoźniak, MarcinKristak, LubosWu, ZhengyanKuchler-Bopp, SabineWychowański, PiotrKumar, AnujWyffels, FrancisKuo, Yong-LinXiao, LiLamas-Galdo, María IsabelXu, FengyuLan, Ting-HsunXu, JingLarabi-Marie-Sainte, SouadXu, LinsenLe Ferrand, HortenseXu, Nicole W.Lee, Lai SoonYamamoto, MakotoLevashenko, VitalyYang, Chung-WeiLi, DechangYang, GuangLi, JiannanYang, Lung-JiehLi, JiehaoYarali, EbrahimLi, JiushengYeh, Szu I.Li, LiangYu, JiaquanLi, ShiguoYücesoy, Deniz TanılLi, ShuaiZakaria, Mohd NormaniLi, WanboZglobicka, IzabelaLiang, HaizhaoZhang, BinLiang, LihongZhang, RuiLiang, TingZhang, XiaoLiauzun, CedricZhang, XiuliLin, ShudongZhao, ChangshengLin, Yu-ChihZhao, GuoruLiu, ChengjuZhao, Yaoyao FionaLiu, YanweiZheng, JianfengLiu, YingtaoZhong, YongLiu, YujiongZhou, XiaodongLiu, ZengqianZhou, YinggeLopresti, FrancescoZou, Xiang-JunLukács, PeterZyla, GordonMa, Yifan



